# Is immunohistochemistry of BRAF V600E useful as a screening tool and during progression disease of melanoma patients?

**DOI:** 10.1186/s12885-016-2951-4

**Published:** 2016-11-18

**Authors:** Laura Schirosi, Sabino Strippoli, Francesca Gaudio, Giusi Graziano, Ondina Popescu, Michele Guida, Giovanni Simone, Anita Mangia

**Affiliations:** 1 Functional Biomorphology Laboratory, IRCCS Istituto Tumori “Giovanni Paolo II”, Viale Orazio Flacco 65, 70124 Bari, Italy; 2 Medical Oncology Department, IRCCS Istituto Tumori “Giovanni Paolo II”, Viale Orazio Flacco 65, 70124 Bari, Italy; 3 Scientific Direction, IRCCS Istituto Tumori “Giovanni Paolo II”, Viale Orazio Flacco 65, 70124 Bari, Italy; 4 Pathology Department, IRCCS Istituto Tumori “Giovanni Paolo II”, Viale Orazio Flacco 65, 70124 Bari, Italy

**Keywords:** Melanoma, BRAF, VE1, Immunohistochemistry, Progression

## Abstract

**Background:**

In clinical practice the gold standard method to assess *BRAF* status in patients with metastatic melanoma is based on molecular assays. Recently, a mutation-specific monoclonal antibody (VE1), which detects the BRAF V600E mutated protein, has been developed. With this study we aimed to confirm the clinical value of the VE1 Ventana® antibody, as today a univocal validated and accredited immunohistochemical procedure does not exist, to preliminary detect BRAF status in our routine diagnostic procedures. Moreover, we explored the biological meaning of BRAF immunohistochemical labeling both as a predictor marker of response to target therapy and, for the first time, as a player of acquired tumor drug resistance.

**Methods:**

We analyzed a retrospective series of 64 metastatic melanoma samples, previously investigated for molecular *BRAF* status, using a fully automatized immunohistochemical method. We correlated the data to the clinicopathologic characteristics of patients and their clinical outcome.

**Results:**

The sensitivity and the specificity of the Ventana® VE1 antibody were 89.2 and 96.2% respectively, while the positive predictive value and negative predictive value were 97.1 and 86.2%, respectively. For six mutated patients the histological sample before treatment and when disease progressed was available. The immunohistochemical BRAF V600E expression in the specimens when disease progressed was less intense and more heterogeneous compared to the basal expression. Multivariate analysis revealed that a less intense grade of positive expression is an independent predictor of a less aggressive stage at diagnosis (*p* = 0.0413).

**Conclusions:**

Our findings encourage the introduction of immunohistochemistry as a rapid screening tool for the assessment of BRAF status in melanoma patients in routine diagnostic procedures and prepare the ground for other studies to highlight the role of immunohistochemical BRAF V600E expression in patients at the time of progression.

## Background

Melanoma is a challenging malignancy to treat, and with its increasing incidence is the fifth and the seventh most common cancer diagnosed in men and women respectively [1]. About 40–60% of cutaneous melanomas have *BRAF* mutations and 90% of these involve a specific missense substitution of valine by glutamic acid at codon 600 (V600E). This mutation constitutively activates the protein and the downstream MAPK signaling pathway in a RAS-independent manner, promoting proliferation, survival and spreading of tumor cells [2]. Metastatic melanoma patients harboring this hot spot mutation can be effectively treated with BRAF inhibitors alone or in combination with MEK inhibitors [3, 4] because this genetic alteration is predictive to therapeutic response. Therefore, rapid screening for *BRAF* status in patients with unresectable or metastatic melanoma has recently become integral to treatment decisions and essential for optimal patient care. In clinical practice the gold standard and the most commonly used method to assess *BRAF* status is based on DNA molecular assays. The most common ones are the classical Sanger sequencing, pyrosequencing and the FDA-approved cobas® 4800 *BRAF V600* mutation test. Each method has its own sensitivity, specificity, cost and response delay [5, 6]. However, the molecular methods are often more time consuming and not always routinely available in all anatomic pathology laboratories. Moreover, some diagnostic samples are still unsuitable for molecular testing because of their inadequate tumor content and the variable quality of DNA extracted due to fragmentation that occurs with tissue processing. The effect of melanin pigment on molecular assays is also important. Thus, there are circumstances when the alternative diagnostic *BRAF* mutation detection method may have utility [7]. Recently, a mutation-specific mouse monoclonal antibody (clone VE1), which does not detect other mutant BRAF V600 epitopes or the wild type form but only the BRAF V600E mutated protein, has been developed [8] and it is now commercially available from Spring Bioscience and Ventana®. It has been previously shown that immunohistochemistry (IHC) with this antibody is sensitive and specific for the detection of the genomic BRAF V600E mutation [9–11]. This finding has permitted the use of IHC, which is a potentially faster, less expensive, and more available methodology to assess BRAF status in the formalin-fixed and paraffin-embedded tissue of melanoma patients [2].

The aim of our study was to confirm the clinical value of the VE1 Ventana® antibody as we plan to use an immunohistochemical method to preliminary detect BRAF status in our routine diagnostic procedures. We intend to use a fully automatized immunohistochemical method, marked CE-IVD, to analyze a retrospective series of metastatic melanoma samples previously investigated for *BRAF* genetic status by molecular techniques and to compare the results obtained by the two methods, as today a univocal validated and accredited immunohistochemical procedure and criteria for analysis does not exist.

Moreover, we propose to explore the biological meaning of BRAF immunohistochemical labeling both as a marker predictive of response to target therapy and as a player of acquired tumor drug resistance. Therefore, we intend to correlate the data to the clinicopathologic characteristics of patients and their clinical outcome, and to compare, in a small subset of patients, BRAF labeling before treatment and when disease progressed.

## Methods

### Patients and samples

Our retrospective study included 64 patients enrolled from June 2008 to April 2015 with histologically confirmed metastatic melanoma treated at the *IRCCS Istituto Tumori “Giovanni Paolo II”* of Bari, with a median age at diagnosis of 61 years (range 22–82 years); 35 patients were male (54.7%) while 29 were female (45.3%). Clinical and follow-up data were collected and evaluated in the entire set of patients, according to approval by the local Ethics Committee of the *IRCCS Istituto Tumori “Giovanni Paolo II”* of Bari (prot. no. 515/EC of May 12, 2015). All patients signed informed consent form authorizing the Institute to utilize biological materials for research purpose according to ethical standards. The study was conducted in accordance with the international standards of good clinical practice. The immunohistochemical analysis of BRAF status was conducted after the end of treatment and, thus, did not influence any therapeutic choice. Regarding the tested samples, five were primary melanoma while the others were principally subcutaneous metastasis (25/64 cases) and lymph node metastasis (21/64 cases), followed by metastasis in other anatomical sites such as the gut, brain, liver (*n* = 13). In all cases the assessment of *BRAF* status had been previously performed by some molecular techniques introduced over the years in the genetic laboratory: for 29 cases the cobas® BRAF V600 test was used, in 22 cases Sanger sequencing was applied, and for the other samples pyrosequencing (13 cases). Forty-two cases were *BRAF* mutated at codon 600 (V600E in 37 patients and V600K in five patients), whereas 22 patients were *BRAF* wild type. The clinicopathological characteristics of the overall cohort and BRAF inhibitor treated patients are shown in Table 1. The site of primary melanoma was the skin in 54 patients, unknown in eight and uveal in two cases. Only one case had stage I disease at diagnosis, 19 (30.1%) patients were at stage II, 26 (41.3%) were at stage III and 17 (27%) at stage IV. According to the American Joint Committee on Cancer (AJCC) melanoma staging system, 13 patients (20.3%) had M1a disease, nine patients (14.1%) had M1b disease and 42 (65.6%) had M1c stage. Twenty-three patients (35.9%) had brain metastasis and 36 (56.3%) had more than two metastatic sites. We also considered Disease Free Survival (DFS), as the time (in months) from diagnosis to the date of the first metastasis and the median was of 12 months (range 0–144). Overall Survival (OS) was defined as the time (in months) from diagnosis to the date of last contact or of death from any cause and the median OS resulted of 32 months (range 2–182), while the median value of OS from metastatic disease (OSMD) to the date of last contact or of death from any cause was 12.5 months (range 2–86).

**Table 1:** Clinicopathological characteristics of overall and BRAF inhibitor treated patients

Characteristics	Overall		BRAF inhibitor treated	
	*N*	(%)	*N*	(%)
Patients	64	(100)	33	(100)
Sex				
Male	35	(54.7)	17	(51.5)
Female	29	(45.3)	16	(48.5)
Age				
Median (range)	61 (22–82)	─	53 (22–82)	─
Site of primary melanoma				
Skin	54	(84.4)	29	(87.9)
Unknown	8	(12.5)	4	(12.1)
Uveal	2	(3.1)	0	(0)
Stage at diagnosis				
I	1	(1.6)	1	(3.1)
II	19	(30.1)	8	(24.2)
III	26	(41.3)	14	(42.4)
IV	17	(27)	10	(30.3)
Metastatic stage				
M1a	13	(20.3)	7	(21.2)
M1b	9	(14.1)	6	(18.2)
M1c	42	(65.6)	20	(60.6)
Brain metastasis				
No	41	(64.1)	23	(69.7)
Yes	23	(35.9)	10	(30.3)
N° metastasis				
≤ 2	28	(43.7)	13	(39.4)
> 2	36	(56.3)	20	(60.6)
DFS				
Median (range)	12 (0–144)	─	12 (0–136)	─
OS				
Median (range)	32 (2–182)	─	31.5 (5–145)	─
OSMD				
Median (range)	12.5 (2–86)	─	14 (4–54)	─

*DFS* disease free survival, *OS* overall survival, *OSMD* overall survival from metastatic disease

For one patient the stage at diagnosis was unknown

Among the BRAF mutated patients, nine were never treated: in seven cases because of a sudden fall in performance status which led to death and in two cases due to a metastasectomy which left them without evidence of disease. The remaining 33 (51.6%) patients of this metastatic melanoma population were treated with BRAF inhibitors. Eighteen patients were treated as first line therapy, 10 as second line and five as third line. Twenty-six patients (78.8%) were treated with Vemurafenib, and seven patients (21.2%) with Dabrafenib at the standard dose of 960 mg and 150 mg respectively twice daily until progression. In this subset of patients we compared also BRAF inhibitor treatment outcome to the immunohistochemical staining. Patients were selected if they had measurable lesions; adequate renal, hepatic and bone marrow functions; an Eastern Cooperative Oncology Group (ECOG) performance status ≤ 2; a life expectancy of more than 12 weeks; and did not need dose reduction or withholdings of doses of BRAF inhibitors for related toxicities.

Patients underwent clinical and laboratory exams every 4 weeks and radiological evaluation with tumor assessments at baseline and then approximately every 12 weeks in order to evaluate therapeutic effectiveness. Response Evaluation Criteria In Solid Tumors (RECIST) version 1.1 was used for efficacy assessment [12]. We assessed the best response during BRAF inhibitors as complete response (CR), partial response (PR), stable disease lasting for at least 12 weeks (SD) and progressive disease (PD). We also measured Progression Free Survival (PFS), defined as the length of time from the start of the treatment until disease progression.

As best response we assessed four CR (12.1%), 22 PR (66.7%) and seven PD (21.2%). The median value of PFS was 6 months (range 1–40) and at the time of the final statistical analysis, conducted in November 2015, all patients had progressed after BRAF inhibitor treatment. For six of these patients the sample at the time of progression was also available and BRAF immunohistochemical analysis was performed also in these specimens to compare the level of expression compared to the basal one.

### Immunohistochemistry

IHC was performed on the same formalin-fixed, paraffin-embedded tissue block used for mutational testing and the histological sections of the relative samples were reviewed by a pathologist to assure the presence of a sufficient tumor content. All specimens were cut into 3–4 μm-thick slices to make sections for immunohistochemical staining using a fully automatized assay based on the Ventana® BRAF V600E (VE1) mouse monoclonal primary antibody on the Ventana® Benchmarck XT (Ventana-Roche Diagnostics, Meylan, France) automated slide strainer in combination with the Ventana *OptiView DAB IHC Detection Kit*®. Briefly, according to the manufacturer’s procedure, after deparaffinization using the *EZ Prep*® reagent, the slides were pretreated with *Cell Conditioning 1®* for 64 min for antigen unmasking and followed by pre-primary antibody peroxidase inhibition. The slides were then incubated with the VE1 antibody at 37 °C for 16 min, and counterstained with *Hematoxylin II*® for 4 min and *Bluing Reagent®* for 4 min. Subsequently slides were removed from the immunostainer, washed in water with a drop of dishwashing detergent and mounted. Negative (a sample wild type for *BRAF* V600E) and positive (a sample with the known V600E mutation) controls were included in each round of analysis. No chromogen was detected when the primary antibody was omitted. All immunoreactive samples were scored by double-blinded independent observers who had no information on patient clinical and molecular data. The results from the two observers were identical in most cases, and the few discrepancies were resolved by re-examination and consensus. The VE1 antibody shows a cytoplasmic staining in positive tumor cells. Immunostaining was primarily interpreted as positive or negative according to Boursault et al. [13]. The slides were scored as positive when more than 90% of tumor cells showed a clear moderate to strong brown cytoplasmic staining, while they were considered negative when there was no staining or only nuclear dot staining, weak staining of single interspersed cells, or staining of monocytes/macrophages. Secondly the intensity of immunostaining was graded 0 if there was no visible staining, grade 1 if weak diffuse cytoplasmic background staining was present, grade 2 if moderate diffuse and granular cytoplasmic staining was observed and grade 3 if strong mainly granular cytoplasmic staining was detected. No staining (grade 0) and staining grade 1 were regarded as negative for V600E, while grade 2 and grade 3 were regarded as positive samples according to Løes et al. [14].

### Statistical analyses

The sensitivity of Ventana® VE1 immunostaining was measured as the proportion of the immunohistochemical positive cases in the molecular assay positive cases, while the specificity was determined as the proportion of the immunohistochemical negative cases in the molecular assay negative cases. Positive predictive value and negative predictive value of the VE1 antibody as compared to molecular analysis were also calculated. Concordance between immunohistochemical expression and molecular analysis was performed using GraphPad QuickCalcs software. A kappa coefficient (k) value of 0.41 to 0.6 indicates moderate agreement, 0.61 to 0.8 substantial agreement and more than 0.8 almost perfect agreement (95% confidential intervals).

Comparison of clinicopathological parameters between the groups of interest (IHC positive and negative, IHC grade 2 and grade 3, mutated and non mutated by molecular techniques, mutated patients treated with BRAF inhibitors and mutated patients non treated) were performed with the Mann Whitney test and the Pearson *χ*2 test or Fisher’s Exact test, when appropriate, for continuous and categorical variables, respectively. The multivariate logistic regression model was used to investigate the effect of some confounding factors on the relation between the outcomes of interest and all the parameters presenting significant association at the univariate analysis.

Survival statistics (DFS, OS, OSMD, PFS) were estimated with the Kaplan-Meier method and the differences between groups of interest was validated by the Log-rank test. The multivariate Cox regression model was used to test for the effect of BRAF status after adjusting for known confounders.

A *p* value < 0.05 was considered significant. All the statistical analyses were performed using SPSS version 17.0 software (SPSS, Inc., Chicago, IL, USA).

## Results

**Fig. 1:**
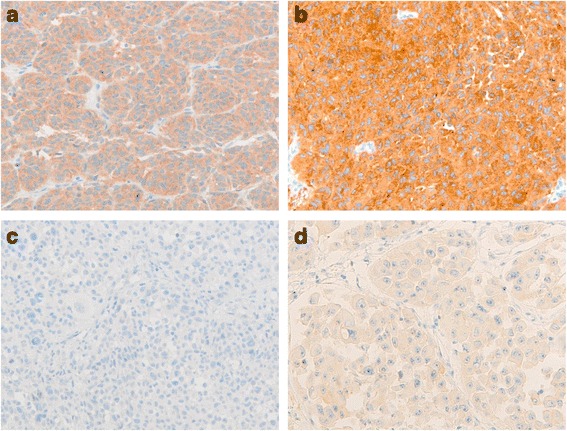
Grade of immunoreactivity of the BRAF V600E Ventana® VE1 antibody in melanoma samples. **a** Moderate diffuse and granular cytoplasmic staining graded as 2. **b** Strong granular cytoplasmic staining graded as 3. **c** Negative staining graded as 0. **d** Weak diffuse cytoplasmic background staining graded as 1. (Original magnification: ×20)

**Fig. 2:**
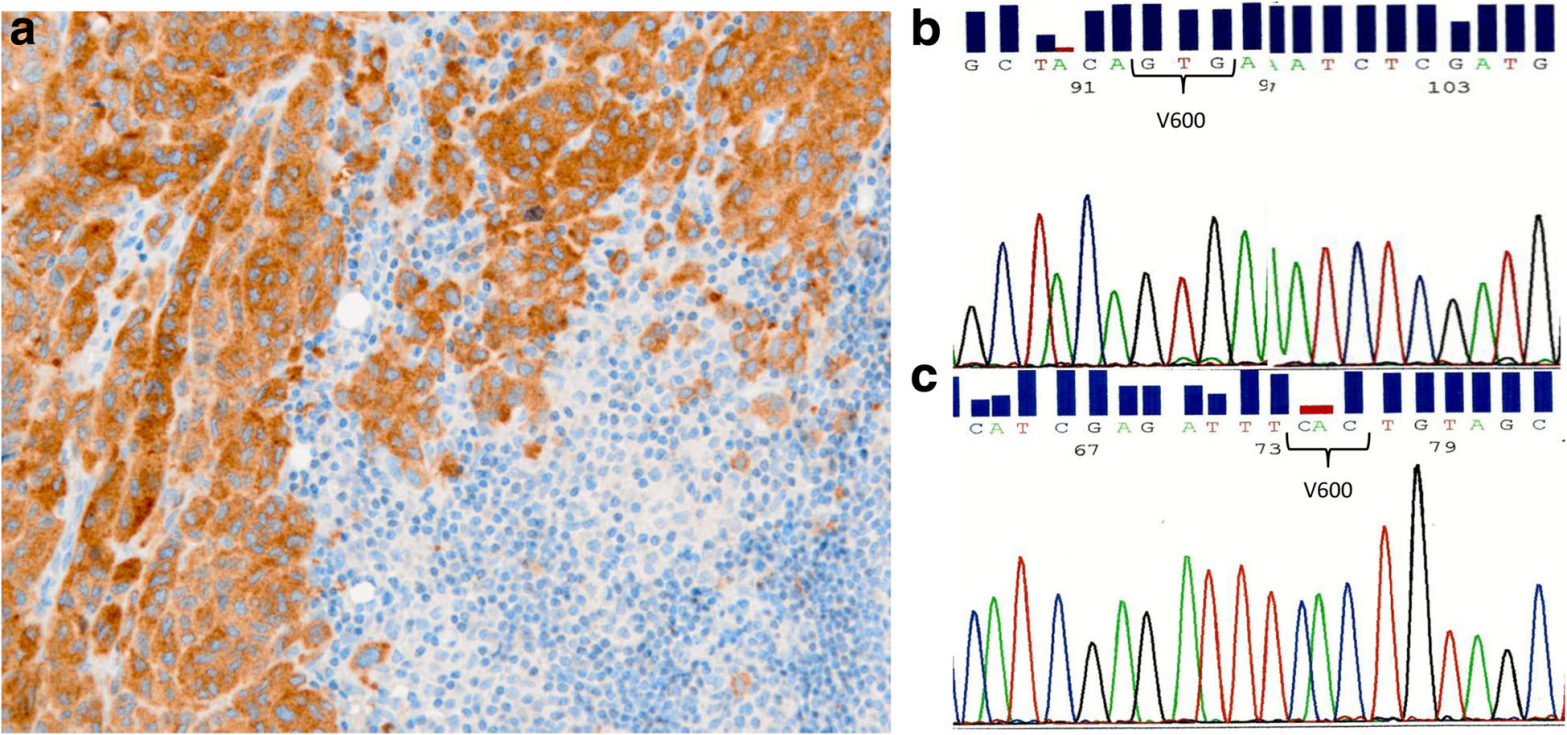
Immunohistochemical and molecular *BRAF* status in a discordant melanoma case. **a** Immunohistochemical BRAF V600E expression graded as three near to an area enriched by lymphocytic normal cells (original magnification: ×20). **b** Sense and **c** antisense sequence of a region of *BRAF* exon 15 in which the V600 codon wild type is underlined

All 64 collected melanoma samples contained a sufficient tumor content to perform immunohistochemical analysis. The Ventana® immunohistochemical assay, using the VE1 mouse monoclonal primary antibody, showed an interpretable result in 63/64 (98.4%) cases. Only one case was not evaluable. It was a subcutaneous metastasis with large, polygonal melanoma cells with very high amount of melanin pigment in cytoplasm and in the surrounding stroma that resulted in nonspecific immunohistochemical reactivity. The samples with the *BRAF* V600E mutation resulted positive in the cytoplasm to VE1 immunostaining, whereas the cases with only the *BRAF* V600K mutation and with the double *BRAF* V600K and *BRAF* V600G mutation remained completely immunonegative. The 34/63 (54%) cases were scored as positive in IHC and showed a moderate to strong brown cytoplasmic staining in more than 90% of tumor cells. In particular, 18 cases resulted grade 2 (Fig. 1a) while the others (*n* = 16) grade 3 (Fig. 1b). The immunoreactivity was almost homogeneous throughout tumor areas and cells, while differences in staining intensity in the analyzed sections were observed in very few cases. The 33/34 (97.1%) immunopositive cases also resulted *BRAF* V600E mutated, confirming what molecular analysis had found. The 29/63 (46%) cases were evaluated as negative: 18 cases were scored as grade 0 (Fig. 1c) while 11 as grade 1 (Fig. 1d). In detail, considering both IHC and molecular techniques 58/63 cases (92.1%) showed concordant results, except five cases (7.9%) which were discrepant even after a second repeated immunohistochemical test. In detail, one case was immunopositive (grade 3) but negative by Sanger sequencing analysis (Fig. 2), while the remaining four cases, which were scored as negative (3 cases were grade 1 and one case was grade 0) in IHC, were *BRAF* V600E mutated when analyzed by Sanger sequencing (2 cases) and the cobas® BRAF V600 test (2 case). Thus, compared to molecular analyses, the sensitivity and the specificity of the Ventana® VE1 antibody were 89.2 and 96.2% respectively, while the positive predictive value and negative predictive value were 97.1 and 86.2%, respectively. Moreover, correlation analysis was performed between immunohistochemical expression and molecular data and resulted in a very good match, since the agreement was 92.1% and k was 0.839. In relation to each single molecular technique, as reported in Table 2, IHC showed a perfect agreement (100%) only with pyrosequencing (k = 1), while the concordance was good (93.1%) with the cobas® BRAF V600 test (k = 0.760) and with Sanger sequencing (85.7%; k = 0.632).

**Table 2:** Correlation between immunohistochemical analysis and molecular methods

		Sanger sequencing		Cobas® test		Pyrosequencing	
		positive	negative	positive	negative	positive	negative
IHC	positive	4	1	23	0	6	0
	negative	2	14	2	4	0	7
Total		6	15	25	4	6	7
Concordance		85.7% (k = 0.632)		93.1% (k = 0.760)		100% (k = 1)	

*IHC* immunohistochemistry

Considering all patients we correlated the immunohistochemical results (negative versus positive samples), the IHC-positive cases (grade 2 versus grade 3), molecular results (mutated versus non mutated patients) and the BRAF-mutated inhibitor treated versus the mutated non treated patients with the following clinicopathologic characteristics: sex, age, stage at diagnosis, metastatic stage of disease, brain metastasis and number of metastasis. We found a statistically significant correlation between the IHC results and sex (*p* = 0.0479) and age (*p* = 0.001) as reported in Table 3. Age was also significantly correlated with molecular results (*p* = 0.0253). The non mutated patients had a median age of 63.64 years while the mutated ones of 53.61 years (data not shown). Kaplan-Meier curves respect to DFS, OS, OSMD showed no statistically significant results. Multivariate analysis, including all the clinicopathologic characteristics as confounding factors, identified grade 2 immunoreactivity as an independent predictor for stage I or II at diagnosis (*p* = 0.0413; Odds Ratio = 11.520; Confidence Interval 1.102 -120.432).

**Table 3:** Correlation of BRAF IHC results and clinicopathological characteristics

	BRAF IHC		
Characteristics	NEGATIVE (*N* = 29)	POSITIVE (*N* = 34)	*p* value
Sex			
Male	20 (68.97)	15 (44.12)	0.0479*
Female	9 (31.03)	19 (55.88)	
Age			
	64.55 ± 12.94	51.18 ± 15.33	0.001*
Stage at diagnosis			
I + II	7 (24.14)	12 (36.36)	0.2975
III + IV	22 (75.86)	21 (63.64)	
Metastatic stage			
M1a	8 (27.59)	5 (14.71)	0.0564
M1b	1 (3.45)	8 (23.53)	
M1c	20 (68.97)	21 (61.76)	
Brain metastasis			
No	20 (68.97)	21 (61.76)	0.5501
Yes	9 (31.03)	13 (38.24)	
N° metastasis			
≤ 2	13 (44.83)	15 (44.12)	0.9549
> 2	16 (55.17)	19 (55.88)	

*p < 0.005

We also considered the subset of patients treated with BRAF inhibitors (*n* = 33), and correlated the negative versus positive IHC results and the IHC-positive samples (grade 2 versus grade 3) with sex, age, stage at diagnosis, metastatic stage of disease, brain metastasis, number of metastases and the best response during BRAF inhibitors. Only age resulted in significant association with IHC results, confirming what had been found in all patients (*p* = 0.0367). Kaplan-Meier curves respect to PFS and multivariate analysis showed no statistically significant results.

With regard to the best BRAF inhibitor response for the discordant cases (*n* = 5), one patient was BRAF V600E by IHC, but he did not receive treatment with BRAF inhibitors on the basis of the wild type molecular result. The OSMD of this patients was of 2 months respect to the median of 12.5 months. Regarding the four cases negative by IHC but mutated by molecular analysis, one patient showed PR, another CR and the last two patients had not been treated.

**Fig. 3:**
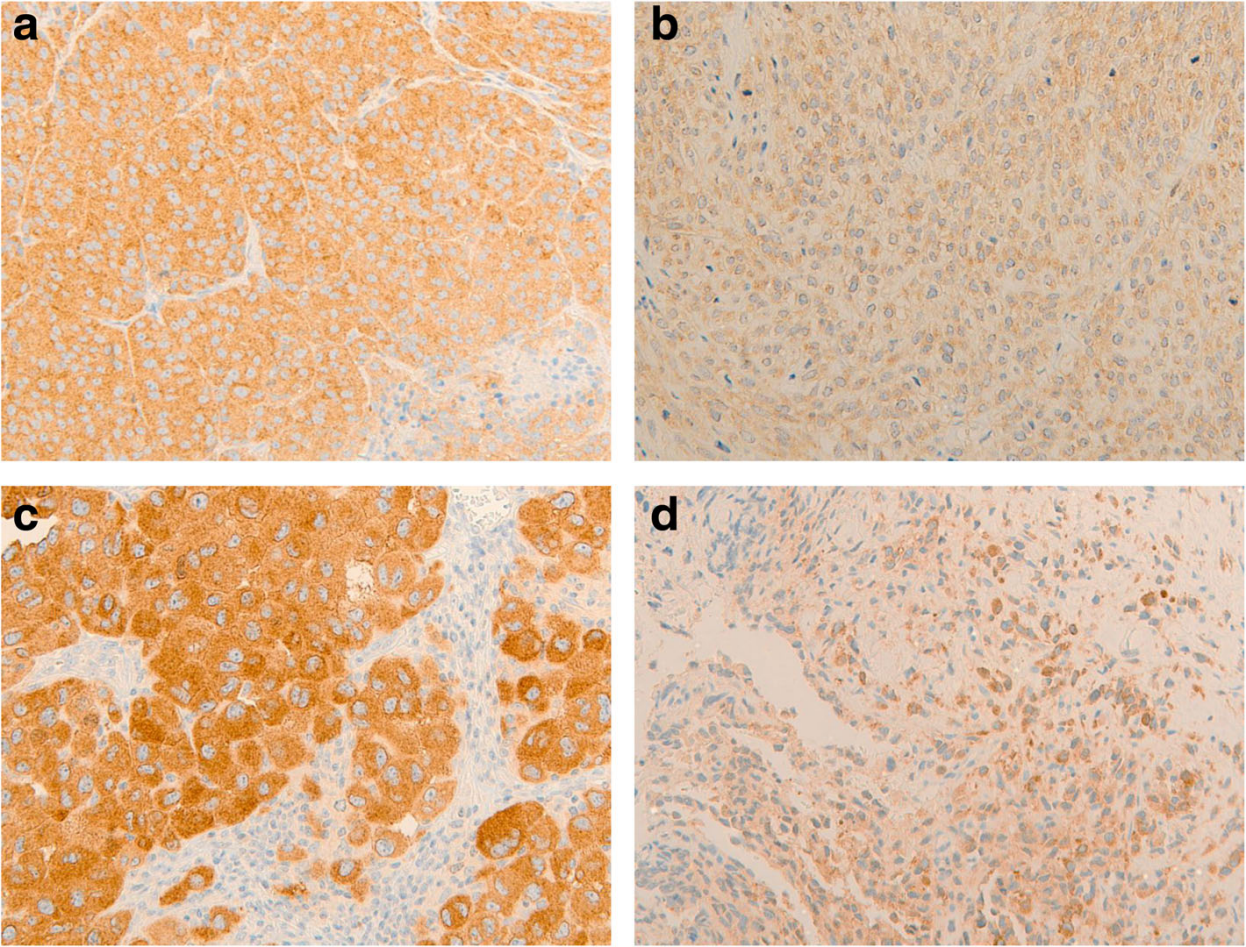
Representative images of BRAF V600E immunohistochemical expression in two patients before treatment and in progression. **a**, **c** In the specimens before treatment we observed a homogeneous BRAF V600E grade 3 immunohistochemical expression. **b**, **d** In the samples when disease progressed we observed a less intense and more heterogeneous BRAF V600E staining. (Original magnification: ×20)

Moreover, for six *BRAF* V600E mutated and treated patients the histological sample before treatment and when disease progressed was available. We analysed immunohistochemical BRAF V600E expression also in these last samples in order to compare the BRAF staining between the pre- and post-treatment specimens. For each patient we noted a difference in BRAF V600E expression between the two samples. In detail, in the specimens when disease progressed we observed a more heterogeneous BRAF V600E staining and areas of neoplastic cells characterized by an intensity of expression weaker than the basal samples, as reported in Fig. 3. However, we not noted any difference among these six patients comparing the post-treatment BRAF V600E staining to their treatment response and PFS.

## Discussion

Immunohistochemistry already covers a central role in the diagnostic pathological setting. In the era of personalized medicine its importance and utility are increasing, since it is a relatively rapid and cheap technique that does not require huge tumor cell content [15]. It is also easy to execute and is considered highly sensitive and specific because the antibodies used, and particularly the mutation-specific monoclonal antibodies, are typically directed against the antigen of therapeutic interest [7, 16]. In this scenario, a new recent monoclonal antibody named VE1 has been developed [8], directed against the mutant BRAF V600E protein, which is the actual target of BRAF inhibitors employed in clinical practice [17].

In our set only one sample resulted not evaluable by immunohistochemical analysis because of the presence of so much melanin that the case was uninterpretable. The Ventana *OptiView DAB IHC Detection Kit*® is a fully automated high-throughput kit that uses 3,3' diaminobenzidine (DAB) as a chromogen of brown intensity. When samples contain an excess of brown tissue pigments, such as melanin, the immunostaining obtained using this kit should be evaluated with caution as it could be a source of interpretative errors [18]. To mitigate for this potential pitfall it is preferable to use a red chromogen, as was carried out in some studies [5, 11, 19–21]. In particular, Thiel et al. [20] referred that melanoma specimens without or with a low content of pigment were easily scored using the DAB chromogen, but in cases with extensive pigmentation successful scoring was obtained using fast Red chromogen. In the near future we are also evaluating the possibility to use another chromogen kit for these particular cases. If this is not available routinely, it is necessary to opt for a different BRAF status detection technique.

The sensitivity and specificity of the Ventana® VE1 antibody found in our study are in line with previous reports that showed them to be in the range of 85 to 100% and 93 to 100% respectively, but these reports used principally the antibody distributed by Spring Bioscience [5, 6, 9, 11, 19, 22]. In particular, considering the study of Qiu et al. [23], who instead used the same fully automatized immunohistochemical method as we did, they found that the sensitivity and specificity of the VE1 antibody were 100 and 99%, respectively. However, it is to consider that in their set there were only 41 cases of malignant melanoma while the majority of them were colorectal carcinomas (611 samples) and papillary thyroid carcinomas (127 specimens) [23]. Our results are close to those of Yaman et al. [24], who, in a study on 48 cases with both primary cutaneous and metastatic melanoma using the fully automatized Ventana® immunohistochemical method with the addition of an amplification signaling kit, reached a positive predictive value of 98.2% and a negative predictive value of 89.7%. More recently Long et al. [25] conducted a study considering 188 metastatic melanoma patients to compare the sensitivity and specificity of the same fully automatized Ventana® immunohistochemical method respect to the pyrosequencing analysis. They obtained a 100% sensitivity and specificity but they considered that the VE1 antibody was positive for BRAF V600E only when strong immunostaining (3+) was observed in at least 80% of tumor cells [25]. Even in our cohort, all grade 3 cases were mutated by molecular analyses, except for the only discordant molecular *BRAF* wild type, but even all grade 2 cases also resulted *BRAF* V600E mutated and in addition three cases graded as 1 and one case graded as 0. In our experience, as supported by other studies [10, 18, 24], also grade 2 staining was associated with mutational *BRAF* V600E status, even if we considered positive the cases with more than 90% of immunoreactive cells. Thus our combination of scoring criteria could be considered valid for immunohistochemical BRAF analysis. Moreover, some false negative results may occur in the case of a long period of cold ischemia and of hypo- or hyper-fixation with formaldehyde [25]. Therefore, in agreement with Long et al. [25], we encourage each laboratory to set the pre-analytical and analytical parameters and to define, by a comparative immunohistochemical-molecular study, the cut-off values in the evaluation of BRAF immunostaining, as today an univocal, validated and accredited immunohistochemical procedure and scoring system for BRAF status does not exist. Our results could therefore be considered useful in this scenario and encouraging to reach this purpose.

**Fig. 4:**
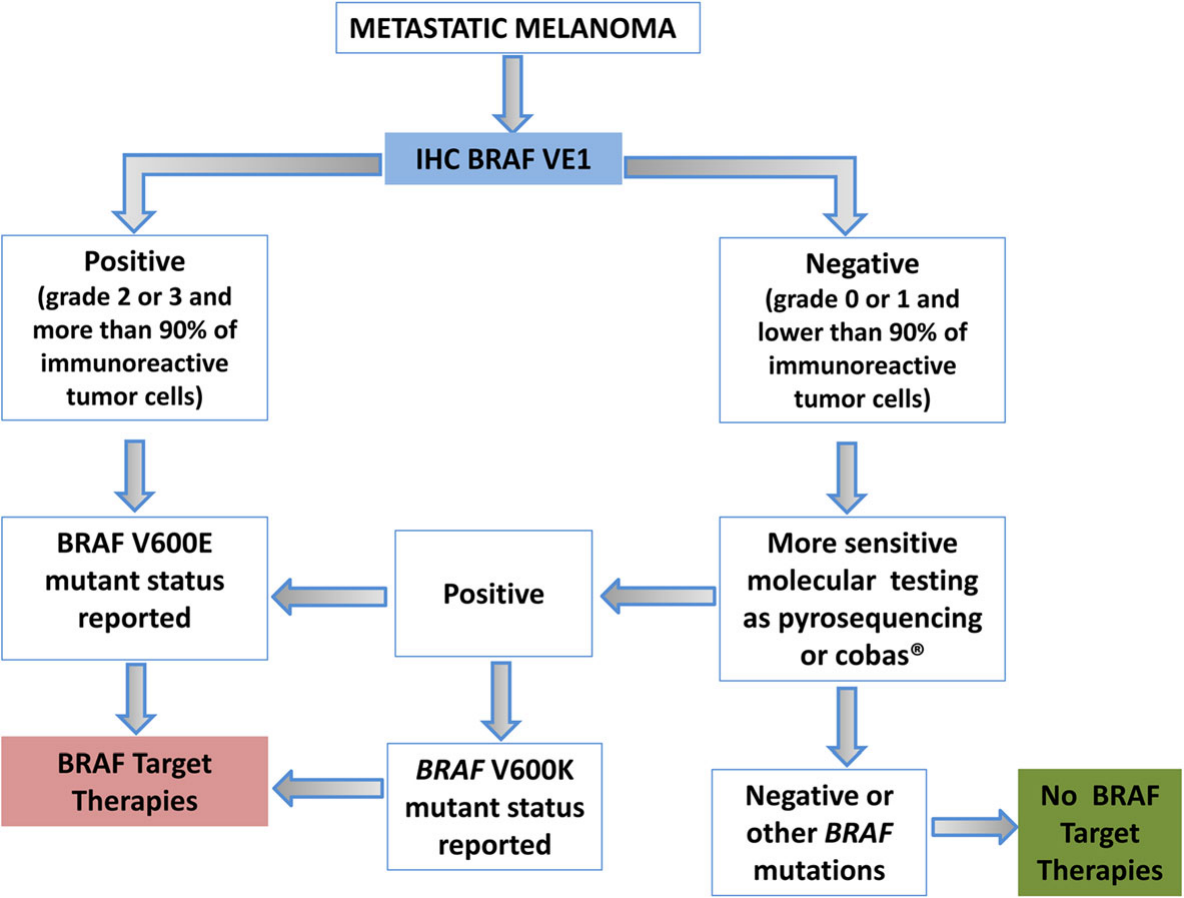
Representative diagnostic algorithm which use BRAF V600E IHC as a screening tool for the selection of patients with metastatic melanoma to be treated with BRAF inhibitors

Moreover, the concordance reached in our study between immunohistochemical expression and molecular data showed a very good match and an almost perfect agreement, even if five discordant cases were recorded. In particular, one case resulted immunopositive but wild type using Sanger Sequencing. This was also reported by Thiel et al. [20], highlighting the importance of performing immunohistochemical staining before DNA extraction for mutational analysis, especially when Sanger Sequencing is used. This molecular technique, in fact, has the lowest sensitivity of the molecular techniques, finding only 20% mutated alleles in a background of wild type alleles [26]. The discrepancy thus could be attributed to the low mutational rate in the DNA extracted or because of intratumoral heterogeneity regarding the presence of BRAF mutated protein [27]. In other studies [9, 24, 28] cases where IHC was positive while a negative result was obtained with a molecular method were also reported, but in some instances the presence of the *BRAF* V600E mutation has been demonstrated with secondary more sensitive molecular techniques. In our case, in the immunohistochemical section we observed the presence of a significant number of normal lymphocytes near the relatively low number of neoplastic cells and there was a very small fraction of non-reactive neoplastic cells. Unfortunately a second DNA extraction in a microdissected area could not be performed and the molecular analysis with other more sensitive methods on the previous extract could not be repeated due to the lack of residual material. The fact that not all mutated *BRAF* V600E samples are IHC-positive, as verified in our other discordant cases, supports the use of DNA mutational analysis only in negative or uncertain patients as they could be a possible immunohistochemical false negative, harboring either a *BRAF* V600E mutation or another *BRAF* mutational variant [21]. In fact, one disadvantage of the use of this antibody in the diagnostic setting is that it is necessary to use an alternative method to detect the other *BRAF* V600 mutations, including in particular *BRAF* V600K. This genetic alteration is the second most common mutation, and it is responsive to dabrafenib, the second BRAF inhibitor currently used in clinical practice [5]. Similar to the majority of other studies [5, 9, 19, 20, 24] we did not detect any cross-reactivity of the VE1 antibody with the BRAF V600K mutated protein, present in five cases. Finally, in our findings IHC showed a perfect agreement with pyrosequencing and a good concordance with the cobas® BRAF V600 test. Pyrosequencing and cobas® are two of the most sensitive molecular methods, able to identify 2% and ≥5% respectively of *BRAF* V600E mutated alleles in a background of wild type alleles [6]. In support of this finding, Colomba et al. [5] proved that pyrosequencing is the most efficient method to detect *BRAF* mutations in melanomas and it should be performed only on VE1-negative or uninterpretable cases. Cobas® test, moreover, is a mutation assay designed to detect *BRAF* V600E mutation, but it is reported also cross-reactivity with *BRAF* V600K [6, 18]. Thus, in our study, the two cases that resulted mutated by cobas® test and negative by IHC could have a mutation different from V600E. Unfortunately this has not been verified by other molecular methods because of the lack of residual material. Our results, supported by the evidence of the literature [5, 11, 19, 24, 25], suggest the use of an algorithm in the melanoma BRAF diagnostic setting (Fig. 4). In this model, IHC should be used in the first instance as a screening tool. The cases resulting VE1 immunonegative should be secondly tested by a DNA mutational assay, using in particular the more sensitive ones to rule out possible false negative samples or those with different *BRAF* mutations. When comparing the cost-effectiveness of IHC and molecular methods there was a strong difference of about 250,00 € between the two assays. Similarly there was a strong difference between the turnaround time, in fact an IHC result was obtained in 24 h whereas the molecular results need of about at least 4–5 days’ work. Thus, this practical algorithm represents the best way in terms of adequacy, rapidity and cost-effectiveness to screen melanoma patients for BRAF V600E mutation.

Regarding the correlation between clinicopathologic characteristics and IHC results, age resulted significantly correlated with BRAF V600E expression. Our finding is in line with literature that reports that the *BRAF* mutation was associated with a younger age of patients [29, 30]. Moreover, the lack of correlation we found with DFS and OS further underlines how the BRAF mutation represents a weak prognostic factor [30]. Considering the subset of treated patients, we also found that the level of expression of BRAF V600E did not predict response or outcome to BRAF inhibitor therapy in metastatic melanoma patients, and these results confirmed what Wilmott et al. [10] had previously found.

This is the first time that IHC has been employed to evaluate BRAF V600E expression in pre- and post-treatment specimens. Despite the restricted number of examined cases, we could suppose that there is a mechanism of resistance which could be linked to the decrease of the level of BRAF V600E expression also accompanied by the heterogeneity of its expression. It could be explained if progression occurs or when resistant tumor subclones expand under the selective pressure of BRAF inhibitors, or as a result of an evolutionary process during treatment, or a combination of both [31]. Considering that in six analyzed cases, regardless the kind of response obtained and the PFS achieved by patients, we found the same pattern of BRAF staining at progression, this biological mechanism of BRAF inhibitor resistance could be relatively common. Thus the analysis of this aspect during the disease progression of mutated patients could be useful in clinical setting management and could support the effort of a combination between BRAF inhibitors and chemotherapy [32]. In our study, we found that a less intense grade of positive expression is an independent predictor of a less aggressive stage at diagnosis. We hypothesize that the intensity of BRAF V600E expression could be correlated to tumor stage aggressiveness at diagnosis in melanoma patients.

## Conclusions

In conclusion, these findings should be confirmed by other studies which could highlight the role of IHC to detect BRAF V600E expression in patients at the time of progression, and to better clarify the meaning of the intensity of positive immunohistochemical expression in melanoma patients. However, on the basis of our experience, we encourage the introduction of IHC as a rapid screening tool for the assessment of BRAF status in melanoma patients in routine diagnostic procedures.

## Abbreviations

AJCC: American Joint Committee on Cancer
CR: Complete response
DAB: 3,3' diaminobenzidine
DFS: Disease Free Survival
ECOG: Eastern Cooperative Oncology Group
IHC: Immunohistochemistry
k: Kappa coefficient
OS: Overall Survival
OSMD: OS from metastatic disease
PD: Progressive disease
PFS: Progression Free Survival
PR: Partial response
RECIST: Response evaluation criteria in solid tumors
SD: Stable disease
